# Novel rechargeable calcium phosphate nanoparticle-containing orthodontic cement

**DOI:** 10.1038/ijos.2016.40

**Published:** 2016-11-04

**Authors:** Xian-Ju Xie, Dan Xing, Lin Wang, Han Zhou, Michael D Weir, Yu-Xing Bai, Hockin HK Xu

**Affiliations:** 1 Department of Orthodontics, School of Stomatology, Capital Medical University, Beijing, China; 2 Biomaterials and Tissue Engineering Division, Department of Endodontics, Periodontics and Prosthodontics, University of Maryland Dental School, Baltimore, USA; 3 Department of Dentistry, China Rehabilitation Research Center, Beijing, China; 4 VIP Integrated Department, Stomatological Hospital of Jilin University, Changchun, China; 5 Center for Stem Cell Biology and Regenerative Medicine, University of Maryland School of Medicine, Baltimore, USA; 6 Department of Mechanical Engineering, University of Maryland, Baltimore County, USA

**Keywords:** bond strength, calcium phosphate nanoparticles, calcium phosphate ion rechargeability, long-term ion release, orthodontic cement, white spot lesions

## Abstract

White spot lesions (WSLs), due to enamel demineralization, occur frequently in orthodontic treatment. We recently developed a novel rechargeable dental composite containing nanoparticles of amorphous calcium phosphate (NACP) with long-term calcium (Ca) and phosphate (P) ion release and caries-inhibiting capability. The objectives of this study were to develop the first NACP-rechargeable orthodontic cement and investigate the effects of recharge duration and frequency on the efficacy of ion re-release. The rechargeable cement consisted of pyromellitic glycerol dimethacrylate (PMGDM) and ethoxylated bisphenol A dimethacrylate (EBPADMA). NACP was mixed into the resin at 40% by mass. Specimens were tested for orthodontic bracket shear bond strength (SBS) to enamel, Ca and P ion initial release, recharge and re-release. The new orthodontic cement exhibited an SBS similar to commercial orthodontic cement without CaP release (*P*>0.1). Specimens after one recharge treatment (e.g., 1 min immersion in recharge solution repeating three times in one day, referred to as “1 min 3 times”) exhibited a substantial and continuous re-release of Ca and P ions for 14 days without further recharge. The ion re-release did not decrease with increasing the number of recharge/re-release cycles (*P*>0.1). The ion re-release concentrations at 14 days versus various recharge treatments were as follows: 1 min 3 times>3 min 2 times>1 min 2 times>6 min 1 time>3 min 1 time>1 min 1 time. In conclusion, although previous studies have shown that NACP nanocomposite remineralized tooth lesions and inhibited caries, the present study developed the first orthodontic cement with Ca and P ion recharge and long-term release capability. This NACP-rechargeable orthodontic cement is a promising therapy to inhibit enamel demineralization and WSLs around orthodontic brackets.

## Introduction

White spot lesions (WSLs) have been reported as a prevailing and challenging problem in fixed orthodontic therapy.^1, 2, 3, 4^ The irregular surfaces of brackets, bands, wires and other attachments provide areas for biofilm/plaque build-up that are difficult to clean.^1, 2, 3^ These conditions promote the colonization of cariogenic bacteria, which produce acids to induce enamel demineralization and can lead to WSLs.^5, 6, 7^ It was reported that 50%–70% of patients with fixed orthodontic therapy had WSLs,^8, 9^ and that it took only 1 month to develop WSLs.^10^ Extensive efforts were made to improve the properties of dental materials, including the use of anti-bacterial agents,^11, 12, 13, 14, 15^ reducing polymerization stresses,^16, 17, 18, 19^ enhancing adhesive systems,^20, 21, 22^ and improving clinical operating methods.^23, 24^ Previous efforts also included methods to combat WSLs in orthodontic treatment.^25, 26, 27^ However, these methods rely on patient compliance and are thus unreliable.^27^

Calcium phosphate (CaP) remineralization approaches may be promising for WSL prevention and enamel remineralization.^28, 29, 30^ Dental resins containing CaP particles were shown to release Ca and P ions.^31, 32^ They act as a Ca and P ion reservoir in plaque and on the tooth surfaces and can be released during an acidic cariogenic challenge to prevent demineralization and facilitate remineralization.^31, 33, 34^ However, traditional CaP composites used CaP particle sizes of 1–55 μm and exhibited poor mechanical properties.^31, 34^ Recently, a novel nanocomposite was developed using nanoparticles of amorphous calcium phosphate (NACP) that had a mean particle size of 116 nm.^35^ The NACP nanocomposite released calcium and phosphate ions similarly to traditional CaP composites, but with a twofold increase in mechanical properties for load-bearing restorations.^35, 36^ The NACP composite could remineralize the enamel lesions, reaching a remineralization efficacy that was fourfold that of a commercial fluoride-releasing composite.^37^ Enamel mineral loss at the margins around the NACP nanocomposite was one-third that of control composite.^38^ However, CaP composites exhibited Ca and P ion release that lasted only a few months before being diminished.^31, 32, 33, 34^

Recently, CaP-rechargeable dental resins were developed for the first time.^39, 40^ NACP was mixed into a resin consisting of pyromellitic glycerol dimethacrylate (PMGDM) and ethoxylated bisphenol A dimethacrylate (EBPADMA).^39^ The specimens were immersed in a pH 4 solution to exhaust Ca and P ion release. Then, the specimens were recharged by immersing them in Ca and P solutions, which could potentially be incorporated into an oral rinse solution.^39^ The recharged specimens were able to re-release high levels of Ca and P ions continuously, without further recharge, for 7 days. After 7 days, the specimens were given a second recharge, and they were able to re-release for 7 more days. Ca and P ions could be repeatedly recharged for re-release, with no decrease in ion re-release while increasing the number of recharge/re-release cycles.^39, 40^ The novel Ca and P ion-rechargeable resins promise to achieve long-term remineralization and caries-inhibiting capabilities. However, the pilot studies only tested one recharge method of 3 min of immersion two times in one day,^39, 40^ without testing other recharge time durations or frequencies. It would be desirable to shorten the immersion time from 3 to 1 min; however, it is not clear if this would compromise the recharge efficacy. Furthermore, to date, there has been no report on the development of a Ca and P ion-rechargeable orthodontic cement to combat WSLs.

Therefore, the objectives of this study were to develop a CaP-rechargeable orthodontic cement and to investigate the effect of recharging time duration and frequency on the recharge/re-release efficacy. The following hypotheses were tested: (1) a new orthodontic cement could be developed to be CaP rechargeable, without decreasing the enamel bond strength; (2) the re-release of Ca and P ions from the orthodontic cement would be maintained over time and would not decrease while increasing the number of recharge/re-release cycles; and (3) the CaP recharge duration and frequency would significantly affect the recharge efficacy of orthodontic cement.

## Materials and methods

### Development of CaP-rechargeable orthodontic cement

The resin consisted of 44.5% (*m/m*) of PMGDM (Hampford, Stratford, CT, USA), 39.5% of EBPADMA (Sigma-Aldrich, St Louis, MO, USA), 10% of 2-hydroxyethyl methacrylate (HEMA) (Esstech, Essington, PA, USA), and 5% of bisphenol A glycidyl dimethacrylate (Esstech, Essington, PA, USA), as described in a previous study.^39^ Then, 1% of phenylbis (2,4,6-trimethylbenzoyl) phosphine oxide (Sigma-Aldrich, St Louis, MO, USA) was added for photopolymerization.^39^ PMGDM and EBPADMA were used because they had cytotoxicity values similar to those of other dental dimethacrylates.^41^ PMGDM is an acidic adhesive monomer^42, 43^ and can chelate with calcium ions from the recharging solution to facilitate ion recharge.^39^ HEMA was added to improve the flowability and hydrophilicity, as reported in a previous study.^44^ BisGMA was added because it could improve the crosslink of monomers and enhance the bonding to tooth structures.^45^ This resin is referred to as PEHB.

NACP [Ca_3_(PO_4_)_2_] were made *via* a spry-drying technique as described previously.^35, 37^ Briefly, calcium carbonate and dicalcium phosphate anhydrous were dissolved in an acetic acid solution. The concentrations of Ca and P ions were 8 and 5.333 mmol·L^−1^, respectively, yielding a Ca/P molar ratio of 1.5. The solution was sprayed into a heated chamber to evaporate the water and volatile acid. The dried NACP was collected using an electrostatic precipitator. The NACP mean particle size was previously measured to be 116 nm.^35^ NACP was mixed with the PEHB resin at an NACP mass fraction of 40% to form the CaP orthodontic cement (referred to as PEHB+NACP). The 40% NACP filler level yielded a flowable paste, with high levels of Ca and P ion release and good mechanical properties, as shown in previous studies.^37, 38^

Transbond XT (3M, Monrovia, CA, USA) is used as an orthodontic cement and served as a control (referred to as the TB control). According to the manufacturer, the TB control consisted of silane-treated quartz (70%–80% by weight), bisphenol A diglycidyl ether dimethacrylate (10%–20%), bisphenol-A-bis (2-hydroxyethyl) dimethacrylate (5%–10%), silane-treated silica (<2%) and diphenyliodonium hexafluorophosphate (<0.2%). Vitremer (3M, St Paul, MN, USA) served as another control and was referred to as the VT control. The VT control is a resin-modified glass ionomer cement (RMGI) that consists of fluoroaluminosilicate glass and a light-sensitive, aqueous polyalkenoic acid. Indications include class III, V and root-caries restorations and class I and II lesions in the primary teeth. VT was selected because RMGIs have been used as orthodontic cements.^46^ A powder/liquid mass ratio of 2.5/L was used for the VT control according to the manufacturer.

### Testing of orthodontic bracket SBS to enamel

Three cements (PEHB+NACP, TB control, VT control) were tested using an enamel shear bond strength (SBS) method.^47^ Sixty extracted human first premolars were randomly divided into three groups of 20 teeth each. The criteria for tooth selection included intact buccal enamel that had not been pre-treated with chemical agents, no visible cracks and no enamel irregularities.^48^ All teeth were stored in 0.01% thymol solution at 4 °C and used within 1 month after extraction. Each tooth was embedded vertically in a self-curing acrylic resin (Lang Dental, Wheeling, IL, USA), considering the buccal axis of the clinical crown, such that their labial surfaces were parallel to the force during the shear bond test.^47^ The coronal portion was submitted to prophylaxis with oil-free pumice and rubber cups at a low speed for 10 s.

For TB control, the buccal surfaces of teeth were etched for 30 s with 35% phosphoric acid (Scotchbond; 3M, St Paul, MN, USA), washed and dried until they acquired a frosty white appearance. Transbond XT primer (3M Unitek, Monrovia, CA, USA) was applied to the etched surfaces in a thin, uniform coat. TB control cement paste was applied to the base of the premolar metal orthodontic brackets (Ormco Series 2000; Sybron Dental, Orange, CA, USA), which was placed on the centre of the tooth surface. Excessive cement around the bracket was removed. The cement was photocured with Optilux VCL 401 (Demetron Kerr, Danbury, CT, USA) for a total of 40 s on all four sides (mesial, distal, occlusal and gingival) of the bracket, with 10 s on each side.^47^ For PEHB+NACP cement, the etching and bonding procedures were the same as those used for the TB control.

For the VT control, according to the manufacturer and the literature, RMGI can be used as an orthodontic cement without etching the enamel.^49, 50, 51^ Therefore, the bonding procedure consisted of polishing the enamel surface for 10 s with flour pumice, followed by rinsing with water for 10 s. Then, the VT paste was applied to the bracket base and the bracket was positioned and bonded to the enamel.^50, 52^ The sample was then light cured for a total of 40 s as described above.^50, 52^

For each of the three groups (PEHB+NACP, TB control, VT control), specimens were divided into two subgroups of 10 samples each. A sodium chloride (NaCl) solution (133 mmol·L^−1^) was prepared and buffered to pH 4 with 50 mmol·L^−1^ lactic acid as a cariogenic, low pH condition.^35^ One subgroup of each cement was immersed in this solution for 1 day, and the other subgroup for 70 days. Thus, a 3 × 2 full factorial design was tested with three cements (PEHB+NACP, TB control, VT control) and two immersion times (1 day, 70 days). Each subgroup of each cement was stored in 50 mL of NaCl solution in a sealed polyethylene container.^53^ For the 70-day subgroups, the solution was changed once every week. At the end of the immersion period, the SBS was measured following previous studies.^51, 54^ A chisel on a Universal Testing Machine (MTS, Eden Prairie, MN, USA) was positioned on the upper part of the bracket base and parallel to the bonded interface. A load was applied at a displacement rate of 0.5 mm·min^−1^ until the bond failed. The SBS=load at failure/bracket surface area, following the previous studies.^47^

Each tested tooth surface was examined using a stereomicroscope (Leica Zoom 2000; Leica, Wetzler, Germany). The adhesive remnant index (ARI) was scored to assess the remaining cement on enamel using the following criteria:^48^ 0=no cement remained on enamel; 1=less than half of the cement remained on enamel; 2=more than half of the cement remained on enamel; and 3=all the cement remained on enamel.

### Measurement of initial Ca and P ion release from PEHB+NACP orthodontic cement

The PEHB+NACP cement was tested for Ca and P ion release. The NaCl solution at pH 4 was used to measure the ion release in simulated cariogenic conditions.^35^ As in previous studies,^32, 35^ three specimens of approximately 2 mm × 2 mm × 12 mm were immersed in 50 mL of solution to yield a specimen volume/solution of 2.9 mm^3^·mL^−1^. This was similar to a specimen volume per solution of ~3.0 mm^3^·mL^−1^ reported in a previous study.^32^ The concentrations of Ca and P ions released from the specimens were measured at 1, 3, 5, 7, 14, 21, 28, 35, 42, 49, 56, 63 and 70 days.^35^ At each time period, an aliquot of 0.5 mL was removed and replaced with fresh NaCl solution. The pH of the solution was monitored and adjusted to pH 4 with 50 mmol·L^−1^ lactic acid using a combination pH electrode (Orion, Cambridge, MA, USA).^36^ The aliquots were analysed for their Ca and P ion concentrations *via* a spectrophotometric method (DMS-80 UV-visible; Varian, Palo Alto, CA, USA) using known standards and calibration curves.^35^ This represented the virgin ion release from the cement and was termed “initial release” to differentiate from the subsequent recharge and re-release.

### Recharge of PEHB+NACP orthodontic cement

**Figure 1:**
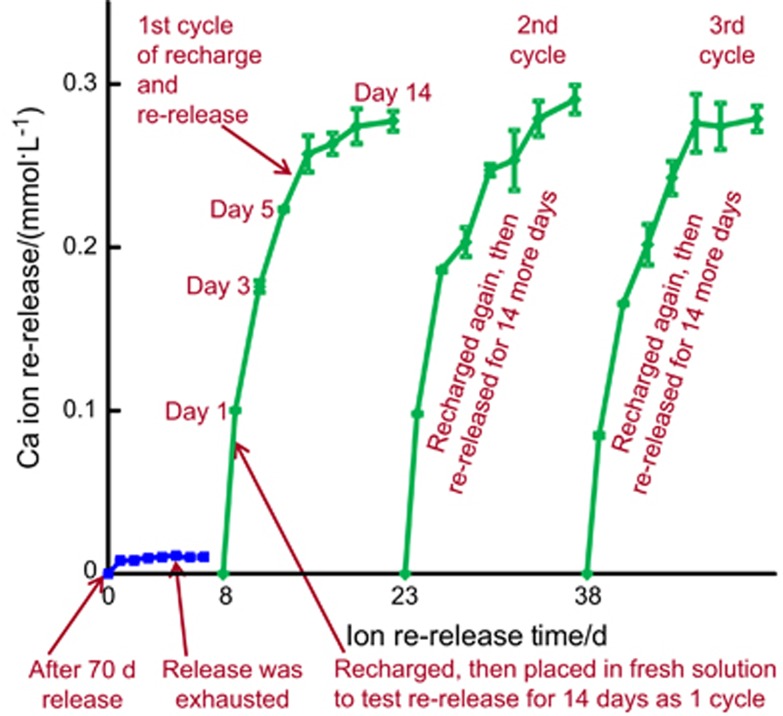
**Illustration of Ca and P ion recharge and re-release**. NACP-containing cement specimens were first immersed in pH 4 solution for 70 days to exhaust the ion release, as indicated by the lower left arrow. Then, the specimens were immersed in fresh pH 4 solution to confirm that the ion release was indeed exhausted, as indicated by the lower middle arrow. The exhausted specimens were recharged in a recharge solution. The recharged specimens were tested for Ca and P ion re-release for 14 days, as indicated by the lower right arrow. This constituted the first recharge/re-release cycle. This was repeated for three cycles. Ca, calcium, NACP, nanoparticles of amorphous calcium phosphate; P, phosphorus.

The procedures of recharge and re-release are illustrated in Figure 1. First, PEHB+NACP cement specimens were immersed in the pH 4 solution for 70 days, as described above for the measurement of initial ion release. The specimens were then removed from the solution and ultrasonicated in distilled water for 30 min. These specimens were denoted “after a 70-day release”, as indicated by the lower left arrow in Figure 1. Then, these specimens were immersed in a fresh pH 4 solution for Ca and P ion measurement for 7 days, which confirmed that indeed the ion release was exhausted and there was no further release, as indicated by the lower middle arrow in Figure 1. Then, these exhausted specimens were used for Ca/P ion recharge. The calcium ion recharge solution consisted of 100 mmol·L^−1^ of CaCl_2_ and 50 mmol·L^−1^ of 4-(2-hydroxyethyl)-1-piperazineethanesulfonic acid (HEPES) buffer.^34, 55^ The phosphate ion recharge solution consisted of 60 mmol·L^−1^ of K_2_HPO_4_ and 50 mmol·L^−1^ of HEPES buffer. Solutions were adjusted to pH 7 using 1 mol·L^−1^ of KOH.^34, 55^ Three specimens of ∼2mm × 2mm × 12 mm were immersed into 5 mL of recharge solution and gently shaken using a mixing machine (Analog Vortex Mixer; Fisher Scientific, Waltham, MA, USA) for 1, 2 or 3 min as listed below. This immersion and shaking treatment represented the movement in the mouth-rinsing process when CaP mouth rinses could be used.^39^ Then, the specimens were rinsed with distilled water to remove any loosely attached deposits on surfaces such that only the ions recharged into the interior of the cement were measured in the subsequent re-release test.

Several different recharge methods were tested. For example, for the recharge using 1-min immersion and repetition three times in one day (referred to as “1 min 3 times”), the aforementioned recharge process was repeated three times at 09:00, 13:00 and 17:00 hours. Then, starting on the next day, the specimens were tested for ion re-release for 14 days continuously (without any recharge during those 14 days). Nine recharge durations and frequencies were tested:

1. 1 min immersion, 1 time a day at 09:00 hours (referred to as “1 min 1 time”).
2. 1 min immersion, 2 times a day at 09:00 and 17:00 hours (referred to as “1 min 2 times”).
3. 1 min immersion, 3 times a day at 09:00, 13:00 and 17:00 hours (“1 min 3 times”).
4. 3 min immersion, 1 time a day at 09:00 hours (“3 min 1 time”).
5. 3 min immersion, 2 times a day at 09:00 and 17:00 hours (“3 min 2 times”).
6. 3 min immersion, 3 times a day at 09:00, 13:00 and 17:00 hours (“3 min 3 times”).
7. 6 min immersion, 1 time a day at 09:00 hours (“6 min 1 time”).
8. 6 min immersion, 2 times a day at 09:00 and 17:00 pm (“6 min 2 times”).
9. 6 min immersion, 3 times a day at 09:00, 13:00 and 17:00 (“6 min 3 times”).

### Dye-assisted recharge solution testing

As an initial screening test of the efficacy of the nine recharge methods, rhodamine B fluorescent dye (Aldrich, Milwaukee, WI, USA) was dissolved in the calcium recharge solution at a concentration of 1%.^56^ The exhausted specimens were immersed in this recharge solution with rhodamine B fluorescent dye. After each of the nine recharge treatments, each specimen was cut in the middle to expose its 2 mm × 2 mm cross-section using a diamond saw (Isomet, Buehler, Lake Bluff, IL, USA). Each cross-section was examined using an epifluorescence microscope (Eclipse TE2000-S; Nikon, Melville, NY, USA) to examine the dye penetration depth into the specimen.^56^

### Measurement of Ca and P ion re-release from recharged PEHB+NACP cement

A preliminary dye-assisted recharge solution test showed that the penetration depths in cement specimens were statistically the same for these four recharge treatments: 1 min 3 times, 3 min 3 times, 6 min 2 times and 6 min 3 times. The 3 min 3 times, 6 min 2 times and 6 min 3 times treatments took longer to perform but exhibited the same recharge efficacy as 1 min 3 times. Therefore, 1 min 3 times was selected from these four treatments. The 3 min 3 times, 6 min 2 times and 6 min 3 times treatments were not included in subsequent tests owing to longer recharge times with no significant benefit.

Therefore, six recharge treatments were tested for Ca and P ion re-release: (1) 1 min 1 time; (2) 1 min 2 times; (3) 1 min 3 times; (4) 3 min 1 time; (5) 3 min 2 times; and (6) 6 min 1 time. The recharged specimens were immersed in 50 mL of pH 4 solution to measure the Ca and P ion re-release using the method described above, as indicated by the third arrow in the bottom of Figure 1. The re-release was tested for 14 days continuously, named cycle 1. After 14 days of re-release, the specimens received the second recharge and were then tested for re-release continuously for 14 days, named cycle 2. This was repeated for three cycles in the present study, as illustrated in Figure 1.

### Statistical analysis

The Kolmogorov–Smirnov test and Levene's test were performed to confirm the normality and equal variance of the data. The results of SBS, recharge solution diffusion depth and Ca/P ion release/re-release were analysed with two-way analyses of variance. *Post hoc* multiple comparisons were performed using the Tukey's honestly significant difference test. The results of ARI were evaluated using the *χ*^2^ test. Statistical significances were preset at *P*<0.05 using the SPSS 14.0 software package (SPSS, Chicago, IL, USA).

## Results

**Figure 2:**
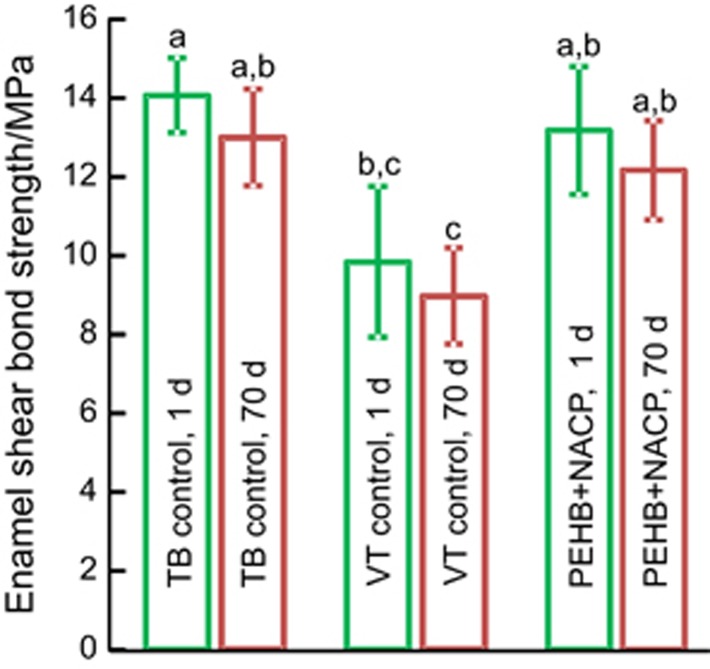
**Enamel shear bond strengths of orthodontic cements after immersion in pH 4 solution for 1 day or 70 days.** Different letters indicate significant differences (*P*<0.05). Mean±standard deviation, *n*=10. NACP, nanoparticles of amorphous calcium phosphate; TB, Transbond XT; VT, Vitremer.

The orthodontic SBSs to enamel after immersion for 1 or 70 days is plotted in Figure 2 (mean±standard deviation; *n*=10). TB control and PEHB+NACP cement had similar bond strengths (*P*>0.1). VT control had relatively lower bond strengths (*P*<0.05). For each material, there was no significant difference between 1 and 70 days (*P*>0.1). The ARI scores are listed in Table 1. Most of the specimens failed at the bracket–cement interface. VT control had lower ARI scores (*P*<0.05). There was no significant difference between immersion for 1 or 70 days for each cement (*P*>0.1).

**Figure 3:**
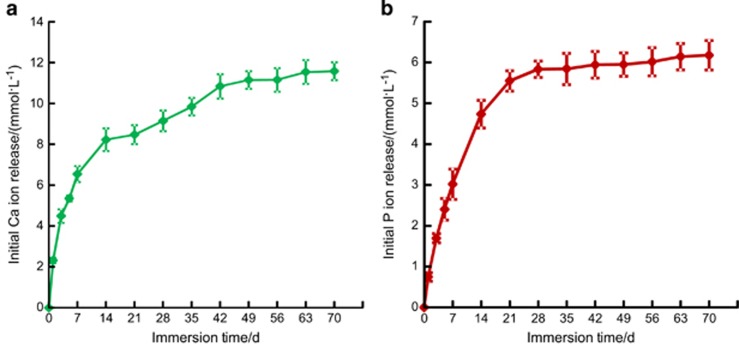
**Initial Ca and P ions release from the PEHB+NACP orthodontic cement.** (**a**) Ca ion; (**b**) P ion. Mean±standard deviation; *n*=6. Ca, calcium; NACP, nanoparticles of amorphous calcium phosphate; P, phosphorus.

The PEHB+NACP cement was tested for initial Ca and P ion release, and the results are shown in Figure 3: (a) Ca ion release and (b) P ion release (mean±standard deviation; *n*=6). The released ion concentrations increased with time from 1 to 35 days; after 35 days, the ion concentration increase was slowed, which indicated relatively less ion release from 35 to 70 days.

**Figure 4:**
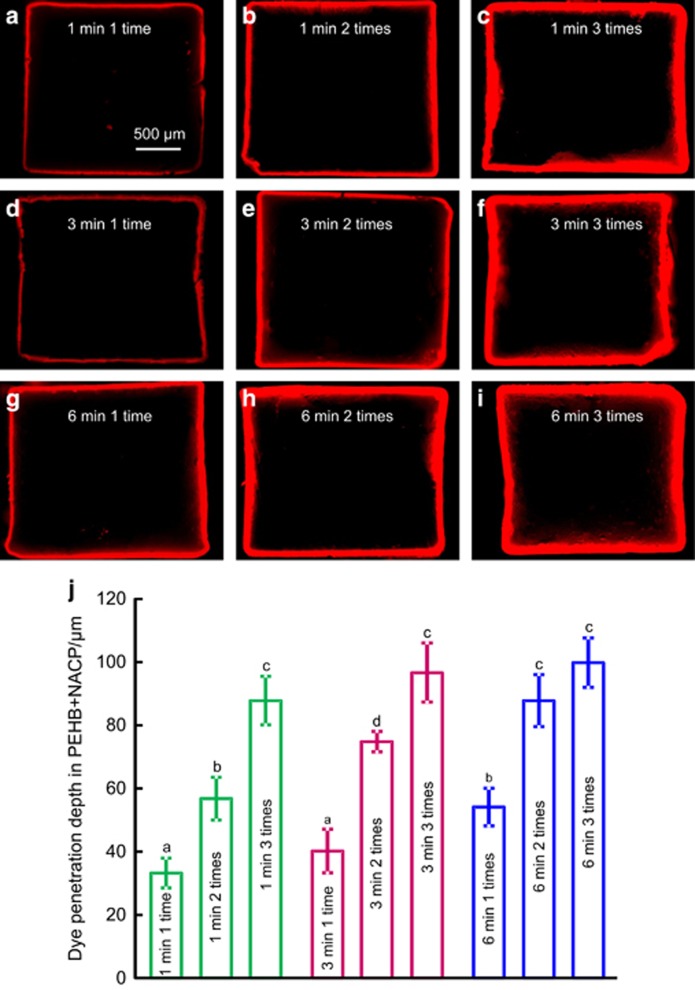
**Typical images of rhodamine B fluorescent dye penetration for different recharge groups.** (**a**) 1 min 1 time; (**b**) 1 min 2 times; (**c**) 1 min 3 times; (**d**) 3 min 1 time; (**e**) 3 min 2 times; (**f**) 3 min 3 times; (**g**) 6 min 1 time; (**h**) 6 min 2 times and (**i**) 6 min 3 times. The image shows the cross-section of the bar of ~2 mm × 2 mm. The red area indicates the depth of penetration. (**j**) Quantitative measurement of the dye penetration depth (mean±standard deviation, *n*=6). Dissimilar letters indicate values that differ from each other (*P*<0.05). NACP, nanoparticles of amorphous calcium phosphate.

Rhodamine B fluorescent images of dye penetration in PEHB+NACP during recharge are shown in Figure 4a–4i. The outer red edge shows the external surface of the specimen, indicating the ~2 mm × 2 mm cross-section of the bar. The thickness of the red line indicates the dye penetration depth, which increased from 1 to 6 min of immersion duration. At each duration, the penetration depth increased from 1 time to 3 times of recharge in a day. The quantitative penetration depth is plotted in (J) (mean±standard deviation, *n*=6). The 1 min 3 times, 3 min 3 times, 6 min 2 times and 6 min 3 times treatments yielded no significant difference (*P*>0.1). When repeating the recharge for 3 times a day, increasing the immersion duration from 1 to 6 min produced no significant increase in penetration depth (*P*>0.1)

**Figure 5:**
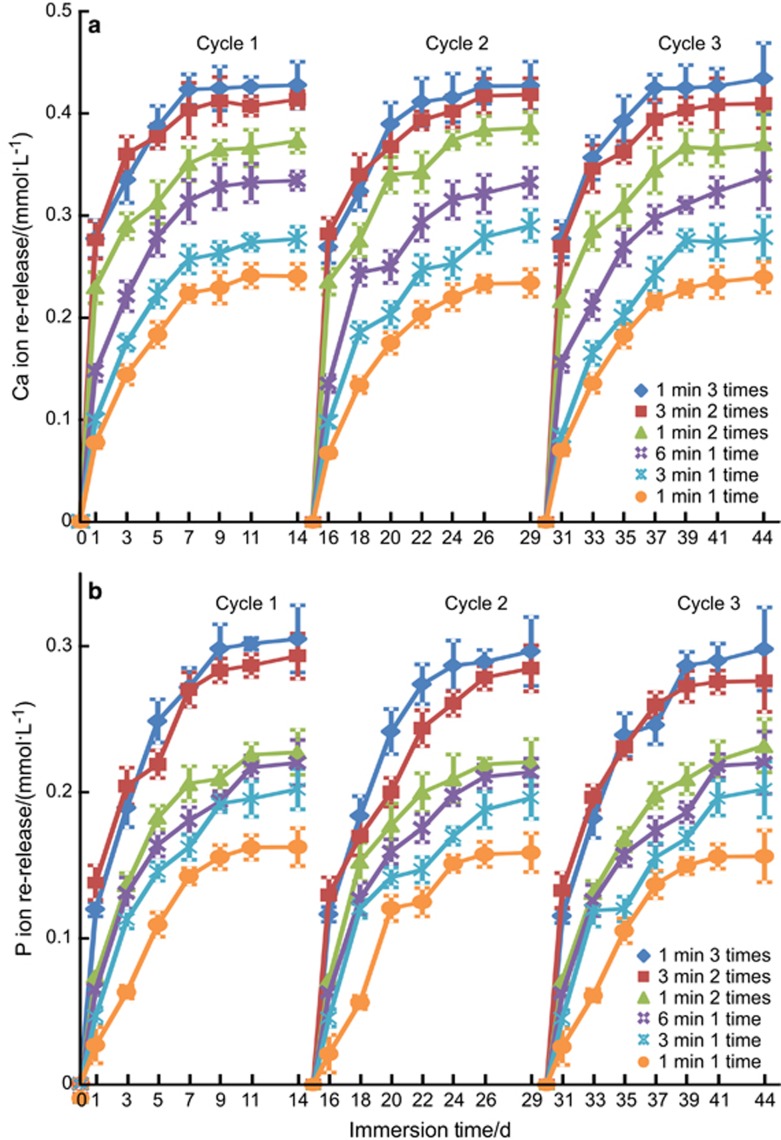
**Re-release from the exhausted and recharged PEHB+NACP orthodontic cement after different recharge treatments**. (**a**) Ca ion; (**b**) P ion. Mean±standard deviation, *n*=3). Three recharge/re-release cycles were tested, and each re-release was measured for 14 days. The ion re-release at 1 min 3 times was the greatest. There was no decrease in re-release when increasing the number of recharge/re-release cycles from 1 to 3 (*P*>0.1). Ca, calcium; NACP, nanoparticles of amorphous calcium phosphate; P, phosphorus.

The PEHB+NACP recharge and re-release results are plotted in Figure 5: (a) Ca and (b) P ion re-release (mean±standard deviation, *n*=3). The recharged specimens were immersed in fresh pH 4 solution and the re-release was measured for 14 days, as one cycle. Therefore, each recharge treatment (which was done in one day) was tested for 14 days of continuous ion release. Three such recharge/re-release cycles were plotted in Figure 5. For all groups, after each recharge treatment, the ion release concentration increased rapidly in the first week, and the release slowed or plateaued in the second week. The ion re-release concentration at 14 days showed the order of 1 min 3 times>3 min 2 times>1 min 2 times>6 min 1 time>3 min 1 time>1 min 1 time. There was no decrease in ion re-release from recharge/re-release cycle 1 to cycle 3.

**Figure 6:**
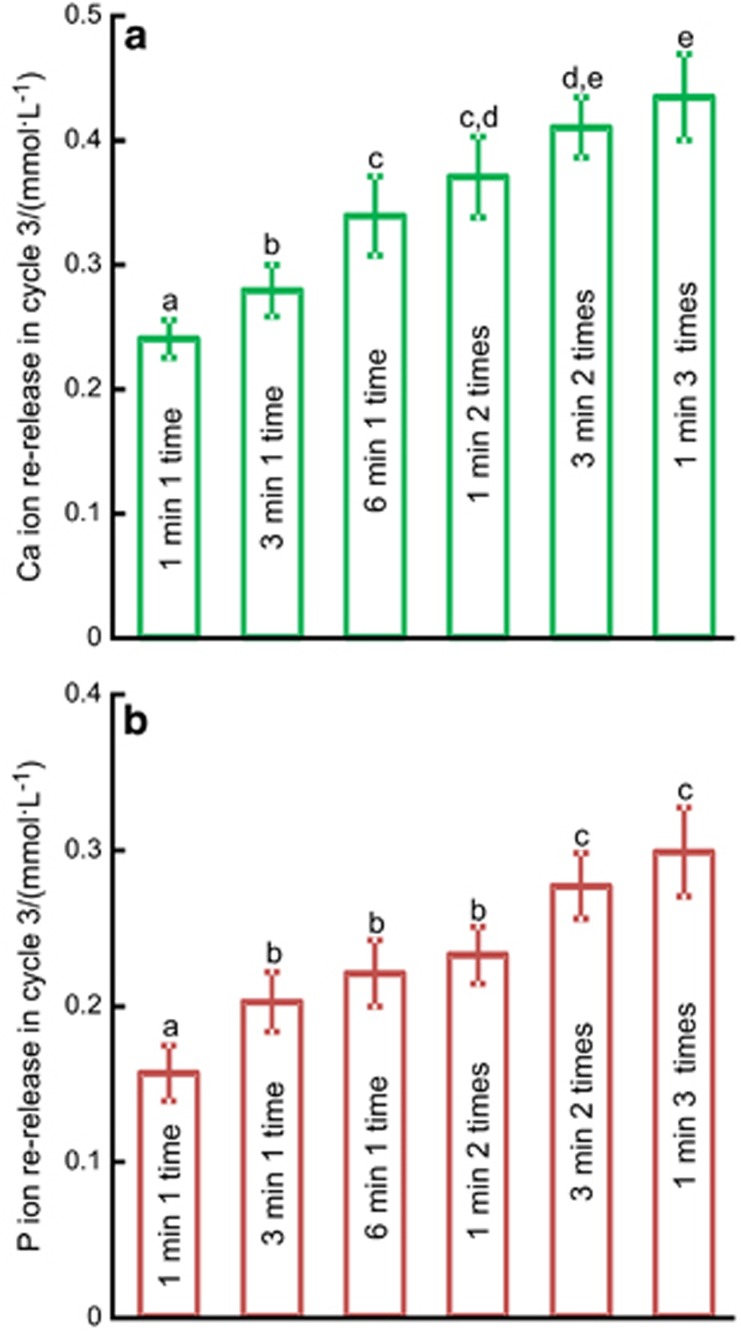
**The Ca and P ion re-release concentrations of different recharge groups at 14 days of the third cycle.** (**a**) Ca ion; (**b**) P ion. Mean±standard deviation, *n*=3. In each plot, dissimilar letters indicate values that are significantly different from each other (*P*<0.05).

For the different recharge treatments, the Ca and P ion concentrations at 14 days of the third recharge/re-release cycle are plotted in Figure 6: (a) Ca and (b) P ion concentrations (mean±standard deviation, *n*=3). The 1 min 3 times treatment had the highest re-release ion concentrations, although it was not significantly different from those of 3 min 2 times (*P*>0.1). However, the 1 min 3 times treatment totalled only 3 min of immersion in recharge, whereas the 3 min 2 times treatment totalled 6 min of immersion in recharge. The 1 min 3 times treatment exhibited significantly higher re-release compared with the first four groups in Figure 6 (*P*<0.05).

## Discussion

An orthodontic cement containing NACP with Ca and P ion recharge and re-release capabilities was developed for the first time. The hypotheses were proven and showed that the new orthodontic cement with CaP recharge did not compromise the enamel bond strength; the re-release of Ca and P ions from the cement was maintained over time and did not decrease while increasing the number of recharge/re-release cycles; further, the recharge immersion time and frequency significantly affected the recharge efficacy of the orthodontic cement. This new orthodontic cement with CaP recharge capability was promising to exhibit long-term Ca and P ion release to combat enamel demineralization and inhibit WSLs during orthodontic treatments.

The recommended bond strengths of metal brackets to enamel were ~8 MPa or greater to provide adequate adhesion to enamel in orthodontic treatments.^57^ In the present study, the bond strength of PEHB+NACP after 24 h of immersion was 13 MPa, similar to that of the TB control and higher compared with that of the VT control. A concern was whether the bond strength of PEHB+NACP would decrease over time because of the release of Ca and P ions. After 70 days of immersion in pH 4 solution, which exhausted the Ca and P ion release, there was no significant decrease in bond strength compared with that at 1 day. The small particle size of NACP likely contributed to the good enamel bond strength, as larger CaP particles in resins could lead to poor mechanical properties for resins with ion release.^31, 34^ Further study is needed to investigate the enamel bond strength of the PEHB+NACP orthodontic cement at times longer than 70 days, such as 1 year and 2 years.

PMGDM and EBPADMA are the major monomers used in the PEHB resin. PMGDM is an acidic adhesive monomer that can chemically chelate with Ca ions because of its carboxylate groups.^42^ EBPADMA is a monomer that is used in dental composites and has a relatively flexible structure, lower vinyl group concentration and lower viscosity than BisGMA systems.^44^ Both HEMA and BisGMA are traditional monomers that are widely used in dental resins.^45^ BisGMA contains ester linkages that connect bisphenol A segments to the polymerizable vinyl segments.^45, 58^ BisGMA has bisphenol A as the core of its chemical structure, which makes it a stiff molecule that produces a mechanically strong polymer. In addition, BisGMA has two pendant hydroxyl groups that can form strong hydrogen bonds with the hydroxyl groups on adjacent BisGMA molecules. Furthermore, the molecular weight of BisGMA is high, which promotes excellent mechanical properties.^45, 58^ However, BisGMA- and HEMA-rich bonding agents may raise the concerns of moderate hydrolysis and the potential degradation of the bonded interface.^59, 60^ Therefore, the quantities of BisGMA and HEMA need to be controlled because they may influence the bonding durability.^61^ The present study used only small amounts of BisGMA and HEMA (5% and 10%, respectively) to minimize any potential negative effect on the bonding durability.

This PEHB resin composition yielded the best CaP rechargeability, as shown previously in comparison with several other resin matrices.^39^ In the present study, this resin yielded a new orthodontic cement with sustained Ca and P ion release, which is promising to inhibit WSL in orthodontic treatments. The rechargeability of PEHB+NACP orthodontic cement likely depend on two mechanisms.^39, 40^ First, the carboxylate groups of PMGDM can chelate with Ca ions in the recharge solution. There likely exists a dynamic equilibrium between the chelation of calcium ions to the PMGDM monomer and the release of calcium ions from the monomer, which is dependent on the local pH of the immersion solution. The solution for recharge (simulating a mouth rinse) had a pH of 7. The PMGDM in the cement may chelate with the calcium ions diffusing from the recharge solution into the resin. After the recharging process, during the re-release, the bond between PMGDM and calcium ions may be severed in the pH 4 solution in which the re-release was measured, simulating a local cariogenic pH from biofilm acids. The second factor that contributed to the recharge may be its space-occupying effect. After the initial Ca and P ion release, which exhausted the ion release, the sites that were previously occupied by the Ca and P ions became available for the incoming Ca and P ions from the recharge solution.

Rhodamine B fluorescent dye has been widely used in bonded interface and microleakage studies.^56, 62^ The deepest layer that the diffusion of the recharge solution reached was ~100 μm as shown in Figure 4j, no matter how long of the total recharge duration was. At 14 days of the third cycle, the 1 min 3 times treatment, with an accumulative total recharge duration of 3 min, released Ca concentration of 0.43 mmol·L^−1^ and P ion concentration of 0.30 mmol·L^−1^. They were higher compared with those of the 6-min, 1-time treatment (with a total recharge duration of 6 min), which yielded a Ca concentration of 0.33 mmol·L^−1^ and P ion concentration of 0.22 mmol·L^−1^. These results indicate that a longer total recharge duration does not always yield a greater ion release, and the recharge efficacy is not directly proportional to the total recharge duration time.

Teenagers may have relatively poor compliance in hygiene and diet controls, but they are the major group of orthodontic patients. Therefore, the CaP recharge procedure for the PEHB+NACP orthodontic cement should be user friendly and require minimal time to perform. Two points should be made. First, the 1 min 3 times a day treatment required a total of 3 min, which is shorter than 6 min 3 times, 6 min 2 times or 3 min 3 times. However, the 1 min, 3 times treatment reached a similar degree of recharge and re-release. Therefore, the 1 min 3 times treatment appeared to be the preferred method. Although it required the 1-min immersion to be repeated three times, this yielded a continuous release of Ca and P ions for at least 7 days. Therefore, the patient could potentially use a mouth rinse for 1 min each after breakfast, lunch and dinner on Sundays to maintain a sustained high level of Ca and P ion release for the whole week. Therefore, the patient only needs to recharge once a week instead of once daily. Second, the re-release was measured with the specimen immersion in a solution with pH 4 continuously as an accelerated test. Clinically, the Stephan curve shows that the plaque pH following glucose intake stays in the cariogenic area of pH 4.5 for <30 min, and then the pH increases back to a safe pH of 6 or higher, after the bacteria have completed their metabolization of glucose and saliva has buffered the acid.^63^ Therefore, instead of being immersed in a pH 4 solution for 24 h every day, the orthodontic cement *in vivo* would likely exhibit a pH close to 4 for only a couple of hours per day, thus preserving the ion reservoir in the cement from being quickly exhausted. This means that after one recharge treatment, the PEHB+NACP orthodontic cement could potentially re-release Ca and P ions for much longer than 2 weeks, perhaps even allowing the patient to undergo recharge treatment (1 min 3 times) only once per month. Further study is needed to investigate the re-release of Ca and P ions from the PEHB+NACP orthodontic cement under pH conditions consistent with the Stephan curve's trend of oral plaque pH, to determine the re-release longevity after each recharge treatment. Further study is also needed to evaluate the inhibition of WSL in enamel using the PEHB+NACP orthodontic cement under simulated conditions *in vivo*.

## Conclusions

In this study, we developed the first orthodontic cement containing NACP with Ca and P ion recharge and re-release capabilities. The new orthodontic cement with Ca and P ion recharge/re-release had an enamel bond strength similar to a commercial cement without CaP release. The NACP orthodontic cement could be repeatedly recharged to maintain the long-term release of Ca and P ions, and the ion release did not decrease when increasing the number of recharge/re-release cycles. The recharge method of 1 min of immersion three times a day appeared to be optimal, yielding the substantial and continuous release of ions for at least 1 week after just one recharge treatment. These results, together with previous studies showing that Ca and P release can effectively inhibit caries and remineralize tooth lesions, suggest that the new rechargeable cement is promising to maintain long-term Ca and P ion release to inhibit enamel demineralization and to avoid WSLs in orthodontic treatments.

## Figures and Tables

**Table 1:** ARI scores of orthodontic cements

		ARI scores (*n*=10)*				
Group	Solution aging	0	1	2	3	SIG^†^
PEHB+NACP	1 day	2	3	5	0	A
TB control	1 day	1	2	6	1	A
VT control	1 day	3	6	1	0	B
PEHB+NACP	70 days	1	3	6	0	A
TB control	70 days	1	3	6	0	A
VT control	70 days	4	5	1	0	B

ARI, adhesive remnant index; NACP, nanoparticles of amorphous calcium phosphate; SIG, statistical significance; TB, Transbond XT; VT, Vitremer.

ARI: 0=no adhesive left on the tooth surface; 1=less than half of the adhesive was left on the tooth surface; 2=half or more of the adhesive was left on the tooth; 3=the entire adhesive was left on the tooth surface.

† Different letters (A, B) indicating significant differences in the ARI scores (*P*<0.05).

## References

[bib1] Gorton J, Featherstone JDB. *In-vivo* inhibition of demineralization around orthodontic brackets. Am J Orthod Dentofac 2003; 123 (1): 10–14. 10.1067/mod.2003.47 12532056

[bib2] Boersma J, Van der Veen J, Bokhout M et al. Caries prevalence measured with QLF after treatment with fixed orthodontic appliances: influencing factors. Caries Res 2005; 39 (1): 41–47. 15591733 10.1159/000081655

[bib3] Willmot D. White spot lesions after orthodontic treatment. Semin Orthod 2008; 14 (3): 209–219.

[bib4] Julien KC, Buschang PH, Campbell PM. Prevalence of white spot lesion formation during orthodontic treatment. Angle Orthod 2013; 83 (4): 641–647. 23289733 10.2319/071712-584.1 PMC8754044

[bib5] Lovrov S, Hertrich K, Hirschfelder U. Enamel demineralization during fixed orthodontic treatment—incidence and correlation to various oral-hygiene parameters. J Orofac Orthop 2007; 68 (5): 353–363. 17882363 10.1007/s00056-007-0714-1

[bib6] Jurela A, Repic D, Pejda S et al. The effect of two different bracket types on the salivary levels of *S mutans* and *S sobrinus* in the early phase of orthodontic treatment. Angle Orthod 2012; 83 (1): 140–145. 22765642 10.2319/030612-187.1 PMC8805532

[bib7] Lombardo L, Ortan YÖ, Gorgun Ö et al. Changes in the oral environment after placement of lingual and labial orthodontic appliances. Prog Orthod 2013; 14 (1): 1–8. 24326120 10.1186/2196-1042-14-28 PMC4384913

[bib8] Chapman JA, Roberts WE, Eckert GJ et al. Risk factors for incidence and severity of white spot lesions during treatment with fixed orthodontic appliances. Am J Orthod Dentofac 2010; 138 (2): 188–194. 10.1016/j.ajodo.2008.10.019 20691360

[bib9] Tufekci E, Dixon JS, Gunsolley J et al. Prevalence of white spot lesions during orthodontic treatment with fixed appliances. Angle Orthod 2011; 81 (2): 206–210. 21208070 10.2319/051710-262.1 PMC8925248

[bib10] Gorton J, Featherstone JD. *In vivo* inhibition of demineralization around orthodontic brackets. Am J Orthod Dentofac 2003; 123 (1): 10–14. 10.1067/mod.2003.47 12532056

[bib11] Imazato S. Bio-active restorative materials with antibacterial effects: new dimension of innovation in restorative dentistry. Dent Mater J 2009; 28 (1): 11–19. 19280964 10.4012/dmj.28.11

[bib12] Li F, Chen J, Chai Z et al. Effects of a dental adhesive incorporating antibacterial monomer on the growth, adherence and membrane integrity of *Streptococcus mutans*. J Dent 2009; 37 (4): 289–296. 19185408 10.1016/j.jdent.2008.12.004

[bib13] Li F, Weir MD, Chen J et al. Comparison of quaternary ammonium-containing with nano-silver-containing adhesive in antibacterial properties and cytotoxicity. Dent Mater 2013; 29 (4): 450–461. 23428077 10.1016/j.dental.2013.01.012 PMC3631003

[bib14] He L, Deng D, Zhou X et al. Novel tea polyphenol-modified calcium phosphate nanoparticle and its remineralization potential. J Biomed Mater Res B 2015; 103 (8): 1525–1531. 10.1002/jbm.b.33333 25470574

[bib15] Zhang K, Cheng L, Imazato S et al. Effects of dual antibacterial agents MDPB and nano-silver in primer on microcosm biofilm, cytotoxicity and dentine bond properties. J Dent 2013; 41 (5): 464–474. 23402889 10.1016/j.jdent.2013.02.001 PMC3654025

[bib16] Lim B-S, Ferracane J, Sakaguchi R et al. Reduction of polymerization contraction stress for dental composites by two-step light-activation. Dent Mater 2002; 18 (6): 436–444. 12098572 10.1016/s0109-5641(01)00066-5

[bib17] Irie M, Suzuki K, Watts D. Marginal gap formation of light-activated restorative materials: effects of immediate setting shrinkage and bond strength. Dent Mater 2002; 18 (3): 203–210. 11823011 10.1016/s0109-5641(01)00083-5

[bib18] Braga RR, Ballester RY, Ferracane JL. Factors involved in the development of polymerization shrinkage stress in resin-composites: a systematic review. Dent Mater 2005; 21 (10): 962–970. 16085301 10.1016/j.dental.2005.04.018

[bib19] Atai M, Watts DC. A new kinetic model for the photopolymerization shrinkage-strain of dental composites and resin-monomers. Dent Mater 2006; 22 (8): 785–791. 16540163 10.1016/j.dental.2006.02.009

[bib20] Pashley DH, Tay FR. Aggressiveness of contemporary self-etching adhesives. Part II: etching effects on unground enamel. Dent Mater 2001; 17 (5): 430–444. 11445211 10.1016/s0109-5641(00)00104-4

[bib21] Tay FR, Pashley DH, Suh BI et al. Single-step adhesives are permeable membranes. J Dent 2002; 30 (7): 371–382. 12554121 10.1016/s0300-5712(02)00064-7

[bib22] Frankenberger R, Tay FR. Self-etch *vs* etch-and-rinse adhesives: effect of thermo-mechanical fatigue loading on marginal quality of bonded resin composite restorations. Dent Mater 2005; 21 (5): 397–412. 15826696 10.1016/j.dental.2004.07.005

[bib23] Lynch CD, Allen P. Management of the flabby ridge: using contemporary materials to solve an old problem. Brit Dent J 2006; 200 (5): 258–261. 16528326 10.1038/sj.bdj.4813306

[bib24] Lynch CD, Frazier KB, McConnell RJ et al. Minimally invasive management of dental caries: contemporary teaching of posterior resin-based composite placement in US and Canadian dental schools. J Am Dent Assoc 2011; 142 (6): 612–620. 21628682 10.14219/jada.archive.2011.0243

[bib25] Sudjalim T, Woods M, Manton D. Prevention of white spot lesions in orthodontic practice: a contemporary review. Aust Dent J 2006; 51 (4): 284–289. 17256301 10.1111/j.1834-7819.2006.tb00445.x

[bib26] Rogers S, Chadwick B, Treasure E. Fluoride-containing orthodontic adhesives and decalcification in patients with fixed appliances: a systematic review. Am J Orthod Dentofac 2010; 138 (4): 390.e1–390.e8. 10.1016/j.ajodo.2010.02.025 20889037

[bib27] Enaia M, Bock N, Ruf S. White-spot lesions during multibracket appliance treatment: a challenge for clinical excellence. Am J Orthod Dentofac 2011; 140 (1): e17–e24. 10.1016/j.ajodo.2010.12.016 21724067

[bib28] Reynolds EC, Cai F, Cochrane NJ et al. Fluoride and casein phosphopeptide-amorphous calcium phosphate. J Dent Res 2008; 87 (4): 344–348. 18362316 10.1177/154405910808700420

[bib29] Bailey DL, Adams GG, Tsao CE et al. Regression of post-orthodontic lesions by a remineralizing cream. J Dent Res 2009; 88 (12): 1148–1153. 19887683 10.1177/0022034509347168

[bib30] Hamba H, Nikaido T, Inoue G et al. Effects of CPP-ACP with sodium fluoride on inhibition of bovine enamel demineralization: a quantitative assessment using micro-computed tomography. J Dent 2011; 39 (6): 405–413. 21453746 10.1016/j.jdent.2011.03.005

[bib31] Dickens SH, Flaim GM, Takagi S. Mechanical properties and biochemical activity of remineralizing resin-based Ca–PO_4_ cements. Dent Mater 2003; 19 (6): 558–566. 12837405 10.1016/s0109-5641(02)00105-7

[bib32] Regnault WF, Icenogle TB, Antonucci JM et al. Amorphous calcium phosphate/urethane methacrylate resin composites. I. Physicochemical characterization. J Mater Sci Mater Med 2008; 19 (2): 507–515. 17619969 10.1007/s10856-007-3178-3 PMC2391310

[bib33] Skrtic D, Antonucci JM, Eanes ED. Improved properties of amorphous calcium phosphate fillers in remineralizing resin composites. Dent Mater 1996; 12 (5): 295–301. 9170997 10.1016/s0109-5641(96)80037-6

[bib34] Langhorst S, O'Donnell J, Skrtic D. *In vitro* remineralization of enamel by polymeric amorphous calcium phosphate composite: quantitative microradiographic study. Dent Mater 2009; 25 (7): 884–891. 19215975 10.1016/j.dental.2009.01.094 PMC2745073

[bib35] Xu HH, Moreau JL, Sun L et al. Nanocomposite containing amorphous calcium phosphate nanoparticles for caries inhibition. Dent Mater 2011; 27 (8): 762–769. 21514655 10.1016/j.dental.2011.03.016 PMC3125490

[bib36] Moreau JL, Sun L, Chow LC et al. Mechanical and acid neutralizing properties and bacteria inhibition of amorphous calcium phosphate dental nanocomposite. J Biomed Mater Res B 2011; 98 (1): 80–88. 10.1002/jbm.b.31834 PMC3375606 21504057

[bib37] Weir M, Chow L, Xu H. Remineralization of demineralized enamel *via* calcium phosphate nanocomposite. J Dent Res 2012; 91 (10): 979–984. 22933607 10.1177/0022034512458288 PMC3446834

[bib38] Melo MAS, Weir MD, Rodrigues LK et al. Novel calcium phosphate nanocomposite with caries-inhibition in a human *in situ* model. Dent Mater 2013; 29 (2): 231–240. 23140916 10.1016/j.dental.2012.10.010 PMC3561736

[bib39] Zhang L, Weir MD, Hack G et al. Rechargeable dental adhesive with calcium phosphate nanoparticles for long-term ion release. J Dent 2015; 43 (12): 1587–1595. 26144190 10.1016/j.jdent.2015.06.009 PMC5001877

[bib40] Zhang L, Weir MD, Chow LC et al. Novel rechargeable calcium phosphate dental nanocomposite. Dent Mater 2016; 32 (2): 285–293. 26743970 10.1016/j.dental.2015.11.015 PMC5116151

[bib41] Boland EJ, MacDougall M, Carnes DL et al. *In vitro* cytotoxicity of a remineralizing resin-based calcium phosphate cement. Dent Mater 2006; 22 (4): 338–345. 16087229 10.1016/j.dental.2005.05.004

[bib42] Venz S, Dickens B. Modified surface-active monomers for adhesive bonding to dentin. J Dent Res 1993; 72 (3): 582–586. 8383709 10.1177/00220345930720030501

[bib43] Milward PJ, Adusei GO, Lynch CD. Improving some selected properties of dental polyacid-modified composite resins. Dent Mater 2011; 27 (10): 997–1002. 21783240 10.1016/j.dental.2011.06.006

[bib44] Skrtic D, Antonucci JM, Liu DW. Ethoxylated bisphenol dimethacrylate-based amorphous calcium phosphate composites. Acta Biomater 2006; 2 (1): 85–94. 16701862 10.1016/j.actbio.2005.10.004 PMC1839056

[bib45] Van Landuyt KL, Snauwaert J, De Munck J et al. Systematic review of the chemical composition of contemporary dental adhesives. Biomaterials 2007; 28 (26): 3757–3785. 17543382 10.1016/j.biomaterials.2007.04.044

[bib46] Uysal T, Amasyali M, Ozcan S et al. Effect of antibacterial monomer-containing adhesive on enamel demineralization around orthodontic brackets: an *in vivo* study. Am J Orthod Dentofac 2011; 139 (5): 650–656. 10.1016/j.ajodo.2009.06.038 21536208

[bib47] Melo MA, Wu J, Weir MD et al. Novel antibacterial orthodontic cement containing quaternary ammonium monomer dimethylaminododecyl methacrylate. J Dent 2014; 42 (9): 1193–1201. 25035230 10.1016/j.jdent.2014.07.006 PMC4559222

[bib48] Scougall Vilchis RJ, Yamamoto S, Kitai N et al. Shear bond strength of a new fluoride-releasing orthodontic adhesive. Dental Mater J 2007; 26 (1): 45–51. 10.4012/dmj.26.45 17410892

[bib49] Scougall-Vilchis RJ, Ohashi S, Yamamoto K. Effects of 6 self-etching primers on shear bond strength of orthodontic brackets. Am J Orthod Dentofac 2009; 135 (4): 424.e1–424.e7. 10.1016/j.ajodo.2008.12.005 19361722

[bib50] Cheng HY, Chen CH, Li CL et al. Bond strength of orthodontic light-cured resin-modified glass ionomer cement. Eur J Orthodont 2011; 33 (2): 180–184. 10.1093/ejo/cjq056 20805142

[bib51] Poosti M, Ramazanzadeh B, Zebarjad M et al. Shear bond strength and antibacterial effects of orthodontic composite containing TiO_2_ nanoparticles. Eur J Orthodont 2013; 35 (5): 676–679. 10.1093/ejo/cjs073 23264617

[bib52] Tuncer C, Tuncer BB, Ulusoy Ç. Effect of fluoride-releasing light-cured resin on shear bond strength of orthodontic brackets. Am J Orthod Dentofac 2009; 135 (1): 14.e11–14.e16. 10.1016/j.ajodo.2008.09.016 19121495

[bib53] Moreau JL, Weir MD, Giuseppetti AA et al. Long-term mechanical durability of dental nanocomposites containing amorphous calcium phosphate nanoparticles. J Biomed Mater Res B 2012; 100 (5): 1264–1273. 10.1002/jbm.b.32691 PMC3373274 22514160

[bib54] Paschos E, Kleinschrodt T, Clementino-Luedemann T et al. Effect of different bonding agents on prevention of enamel demineralization around orthodontic brackets. Am J Orthod Dentofac 2009; 135 (5): 603–612. 10.1016/j.ajodo.2007.11.028 19409343

[bib55] Meyer-Lueckel H, Hopfenmuller W, Von Klinggraff D et al. Microradiographic study on the effects of mucin-based solutions used as saliva substitutes on demineralised bovine enamel *in vitro*. Arch Oral Biol 2006; 51 (7): 541–547. 16569393 10.1016/j.archoralbio.2006.01.006

[bib56] Banomyong D, Palamara JE, Messer HH et al. Sealing ability of occlusal resin composite restoration using four restorative procedures. Eur J Oral Sci 2008; 116 (6): 571–578. 19049529 10.1111/j.1600-0722.2008.00570.x

[bib57] Reynolds I. A review of direct orthodontic bonding. Br J Orthodont 1975; 2: 171–178.

[bib58] Pashley DH, Tay FR, Carvalho RM et al. From dry bonding to water-wet bonding to ethanol-wet bonding. A review of the interactions between dentin matrix and solvated resins using a macromodel of the hybrid layer. Am J Dent 2007; 20 (1): 7. 17380802

[bib59] Finer Y, Santerre J. The influence of resin chemistry on a dental composite's biodegradation. J Biomed Mate Res A 2004; 69 (2): 233–246. 10.1002/jbm.a.30000 15057996

[bib60] Takahashi M, Nakajima M, Hosaka K et al. Long-term evaluation of water sorption and ultimate tensile strength of HEMA-containing/-free one-step self-etch adhesives. J Dent 2011; 39 (7): 506–512. 21575671 10.1016/j.jdent.2011.04.008

[bib61] Park J, Eslick J, Ye Q et al. The influence of chemical structure on the properties in methacrylate-based dentin adhesives. Dent Mater 2011; 27 (11): 1086–1093. 21816460 10.1016/j.dental.2011.07.011 PMC3190579

[bib62] Profeta A, Mannocci F, Foxton R et al. Experimental etch-and-rinse adhesives doped with bioactive calcium silicate-based micro-fillers to generate therapeutic resin–dentin interfaces. Dent Mater 2013; 29 (7): 729–741. 23639454 10.1016/j.dental.2013.04.001

[bib63] Thylstrup A, Fejerskov O. Textbook of Clinical Cariology. Copenhagen: Munksgaard. 1994.

